# 

**Published:** 1895-07

**Authors:** 


					﻿Vol. XV.
JULY, 1895.
No. 7?
THE
HOMEOPATHIC pHYjSlClAfJ,
A Monthly Journal of Homoeopathic Materia Medica
and Clinical Medicine.
“ He it a freeman whom truth malcet free, and all are tlavet betide."
“Seek the Truth: come whence it may, cost what it will."
EDITED AND PUBLISHED BY
WALTER M. JAMES, M. D.
PHILADELPHIA:
JSTo. 1231 LOCUST STREET.
X»O JT33O-3ST :
ALFRED HEATH <fc CO., Sole Agent# fob Great Britain,
114 Ebury Street, Eaton 8quare.
tCSF Yearly Subteription, $2.50, invariably in advance. Single number^ 25 elf.
Entered at the Philadelphia Fo(t*Offlce at Becond-Claaa Mall Matter.
				

